# Infection prevention and control for diverse vulnerable populations: From an emergency response to the COVID-19 pandemic to sustainable improvement

**DOI:** 10.1177/08404704231198199

**Published:** 2023-09-08

**Authors:** Susan Bisaillon, Sandy Stemp, Krystyna Ostrowska, Melissa Ramprashad, Samantha Herbert

**Affiliations:** 1 8205 Safehaven, Toronto, Ontario, Canada.; 2 Reena, Toronto, Ontario, Canada.; 3 5543 Trillium Health Partners, Mississauga, Ontario, Canada.

## Abstract

At the onset of the COVID-19 pandemic in early 2020, organizations providing residential and respite care for individuals with developmental disabilities and complex care needs in the Greater Toronto Area were largely unprepared. As case numbers surged, they lacked the expertise and resources needed to prevent spread across populations that are highly vulnerable to infection and poor outcomes. This article describes how these organizations, led by Safehaven, responded to an unprecedented emergency, and how the response is leading to sustainable improvements in care and safety for diverse vulnerable groups in congregate care settings. As the pandemic advanced, the Safehaven Program evolved with the solidification of the role of Infection Prevention and Control Champion lead role in Ontario and partnership with Reena in York Region.

## Introduction

In the spring of 2020, Safehaven launched an Innovative Infection Prevention and Control (IPAC) program as an urgent response to the onset of the COVID-19 pandemic.

Safehaven is a registered charity that provides residential, respite, and transitional care for children, youth and adults with developmental disabilities and complex care needs in the Greater Toronto Area (GTA). As COVID-19 case numbers surged, a team of experienced professional healthcare staff, unique within the Developmental Services (DS) sector, identified major gaps in the expertise, experience, standards, and resources needed to protect and care for a highly vulnerable population.

The program initiated by Safehaven incorporates a broad range of IPAC measures. It was developed at Safehaven, offered initially to agencies across the DS sector, and later expanded to include organizations serving diverse vulnerable groups. Although launched as an emergency response, it is evolving into a permanent system of continuously improving disease prevention and care.

## Background: Safehaven

Safehaven^1^ operates six locations in the GTA, serving over 300 families each year. It receives funding from the Ontario Ministry of Children, Community and Social Services (MCCSS) and the Ontario Ministry of Health (MOH).

Safehaven provides year-round, 24/7 care for a wide range of individuals, including those living with cerebral palsy, genetic and seizure disorders, muscular dystrophy, tracheostomy, brain injuries, and other medical complexities.

Safehaven’s approach to care delivery is grounded in a commitment to building inclusive living environments where all clients are treated with dignity and respect. The organization is currently extending services into the community and addressing critical needs such as homecare and affordable housing for DS clients. Partnerships with Toronto area hospitals enable clients who have been living in hospitals for extended periods to transition to Safehaven, freeing up acute and post-acute care capacity.

Safehaven also advocates for those with complex care needs. In 2019, Safehaven launched the “We Belong” movement that changes perceptions of what it means to live with complex care needs, pushes for integration and equity, and communicates a message of inclusion through social and conventional media.

## The challenge: Managing the COVID-19 pandemic in congregate care settings

COVID-19 was officially declared a pandemic by the World Health Organization (WHO) on March 11, 2020. Case numbers surged across the GTA, as in many parts of Canada and globally.

The developmental services sector was largely unprepared. Most DS organizations have no medically trained staff. Congregate care settings present significant infection control challenges, and many clients are highly vulnerable to infection and poor outcomes.^2^ Overall, the sector lacked the expertise and resources to implement measures required to limit the spread of COVID-19 or reduce severe illnesses and deaths.

Safehaven was an exception. A Chief Executive Officer with previous IPAC leadership experience and professional nursing staff education gave Safehaven a unique understanding of IPAC deficiencies, client vulnerability, and the elevated risk of infection in high-density urban living environments.

Safehaven’s initial efforts to address IPAC concerns confronted multiple barriers. Support from the MOH and Toronto Public Health in the first wave of the pandemic was limited, as IPAC resources were focused on crises in hospitals and Long-Term Care (LTC). Pandemic-related staff shortages forced DS organizations to increase the use of costly agency staff. Staffing challenges were further compounded when Ministry directives restricted care providers to working in one organization, forcing DS agencies to compete for nursing and unregulated staff with other sectors that could offer higher salaries, sign-on bonuses, and benefits.

## The solution: A comprehensive IPAC program for congregate care

The particular challenges confronting the developmental services sector prompted multiple forums and discussions. DS organizations across the GTA worked together to support each other and to assume informal leadership roles in key areas such as working to resolve human resources issues and raising awareness of DS sector needs at all levels of government.

Safehaven’s unique healthcare expertise made it a natural fit to assume IPAC leadership for the sector. Safehaven responded by developing a comprehensive IPAC program focused on the needs of vulnerable groups in congregate care settings.

Funding was secured from the Ontario Government COVID Residential Relief Fund (CRRF) through the MCCSS to launch the new program in May 2020. Safehaven’s leadership team assembled an IPAC steering committee, including individuals with expertise in infectious diseases, infection control, nursing, and epidemiology to guide the program and help build IPAC capacity for DS agencies in the GTA.

No precedents or established theoretical frameworks were available to guide the emergency response to the onset of the COVID-19 pandemic in congregate care settings. Safehaven relied upon recognized IPAC experts and authorities for guidance and advice. The leadership team formed connections with specialists from seven local “hub” hospitals for IPAC support, and with Toronto Public Health, Public Health Ontario (PHO), the MCCSS, and Accreditation Canada to ensure alignment with recommended COVID-19 IPAC practices and pandemic response strategies as they evolved.

Program development and implementation required highly focused efforts by DS sector leaders to communicated client needs, to advocate for resources and support, to adapt evolving COVID-19 recommendations for specific sector requirements, to develop strong healthcare and public health partnerships, to enforce adherence to available best practices, and to engage and mobilize staff confronting an unprecedented emergency.

Program components, designed to ensure safety for both clients and staff, include the following:

• Online education modules, adapted to the specific needs of the DS sector and addressing environmental cleaning/disinfection, donning and doffing PPE, hand hygiene, enhanced screening, isolation precautions, outbreak management, and policies and procedures.
• A mobile unit consisting of 25 nurses to deliver COVID-19 testing and swabbing services, environmental inspections, and vaccine clinic support to DS sites.
• Mask-fit testing for frontline staff.
• Regular, cross-regional IPAC collaborative sessions attended virtually by regional DS sector leaders to share information and best practices.
• Epidemiological support to collect data and identify evidence-based opportunities for improvement.
• Regional and provincial coordination through participation in the City of Toronto Accessibility Task Force and the Provincial Network Vaccine Accessibility Committee.

The response to the program from DS organizations was immediate, with high demand for IPAC services and cross-regional collaborative sessions.

In January 2021, as the program spread, the MOH launched an IPAC Champion model to support the DS sector provincially. Safehaven was designated as the IPAC Champion Lead for Toronto. The IPAC Champion model was subsequently expanded to include other sectors funded by the MCCSS, including Youth Justice, Violence Against Women, and Indigenous Health and Healing. Leaders from the Young Infant and Parent sector, and from other organizations providing respite and residential care for children, youth, and adults, also participated in education sessions.

Safehaven also formalized a partnership with Reena, which was designated as the IPAC Champion Lead for York Region. The partnership was successful in further extending IPAC program outreach and helping to ensure cross-regional consistency in IPAC education and practices, a program priority given that many DS agencies have sites in both regions.

In November 2021, Safehaven was awarded a $100,000 grant from the Ontario Federation for Cerebral Palsy. This has been used to purchase a digital learning platform that is enabling IPAC programming to expand across Ontario, including remote communities. Provincial and federal funding has supported the delivery of all content in French and English.

All components of the IPAC program have been aligned with the Ontario Government’s COVID-19 Action Plan for Vulnerable People, which was evolving throughout the pandemic.

## Results: From emergency response to system change

The IPAC programming has been well received across Toronto and York Region. Key benchmarks to date include the following:

• Participation of more than 70 organizations, with more than 600 participating sites;
• Delivery of learning modules to 6,621 attendees from 45 organizations;
• Creation of 2,691 IPAC Champions through additional education;
• 16 COVID-19 vaccine clinics, delivering more than 3,800 COVID vaccinations;
• 27 N95 mask-fit testing clinics;
• 149 COVID-19 swabbing and testing sites;
• 57 IPAC collaborative sessions; and
• 128 flu immunization clinics.

Participating organizations ranged from small agencies with fewer than 100 clients to a large system providing care for more than 4,000 individuals. They included organizations delivering social and educational services to individuals transitioning to community living, as well as organizations focused on providing residential, respite, and transitional care.

Participating staff included both professional and unregulated care providers, senior leaders, and a diverse range of directors, managers, and supervisors.

A survey of education program participants and a series of focus groups were conducted by an independent research firm in 2022 to assess the usefulness of educational modules, the impact on learners, the actions taken within facilities, and recommendations for improvement.^3^ Participants confirmed that the program filled a significant gap within the DS sector, and cited the ability to balance the medical model of health and safety with the community-oriented, home-based model that exists within many DS agencies as a key attribute.

Participants confirmed that the information provided was applicable to DS organizations without modifications and had an impact on changing IPAC practices to improve safety for staff, residents, and families:

• Sixty-five percent of respondents said the sessions taught them new things about IPAC, 62% said they helped them feel better prepared to manage an outbreak in their facility, and 51% said they helped them feel safer in their workplace.
• Seventy-seven percent of respondents reported that they had changed their cleaning or disinfecting practices, 61% made changes to hand hygiene practices, and 57% changed practices for donning and doffing PPE.

A detailed analysis of the direct impact of the IPAC program launched by Safehaven on COVID-19 case numbers is not possible given available data. However, data on confirmed COVID-19 cases from PHO’s Public Health Cases and Contact Management Solution offer general insights into COVID-19 infection rates within congregate care settings.^4^ Data were aggregated by PHO for the period from March 1, 2020, to July 16, 2022, divided into seven COVID-19 waves for Toronto, York, and Ontario. These data were used to compare infection rates in congregate care settings with infection rates in the general population (Table 1).

**Table 1.** Confirmed COVID-19 cases in congregate care/group home settings and the general population.

	Wave 1 - February 26, 2020, to August 31, 2020 (188 days)	Wave 2 - September 1, 2020, to February 28, 2021 (181 days)	Wave 3 - March 1, 2021, to July 31, 2021 (153 days)	Wave 4 - August 1, 2021, to December 14, 2021 (136 days)	Wave 5 - December 15, 2021, to February 28, 2022 (76 days)	Wave 6 - March 1, 2022, to June 18, 2022 (110 days)	Wave 7 - June 19, 2022, to present
Confirmed COVID-19 cases in congregate care/group home settings							
Ontario	495	2,641	1,657	517	3,435	2,820	470
Toronto	163 (32.9%)	633 (24.0%)	329 (19.9%)	43 (8.3%)	494 (14.4%)	141 (5.0%)	33 (7.0%)
York	88 (17.8%)	62 (2.3%)	28 (1.7%)	7 (1.4%)	138 (4.0%)	84 (3.0%)	33 (7.0%)
Toronto/York	251 (50.7%)	695 (26.3%)	357 (21.5%)	50 (9.7%)	632 (18.4%)	225 (8.0%)	66 (14.0%)
Confirmed COVID-19 cases in the general population							
Ontario	42,468	260,625	247,648	87,356	476,870	203,167	32,188
Toronto	14,868 (35.0%)	80,403 (30.9%)	70,832 (28.6%)	15,056 (17.2%)	104,291 (21.9%)	39,138 (19.3%)	7,841 (24.4%)
York	3,448 (8.1%)	25,386 (9.7%)	24,075 (9.7%)	7,107 (8.1%)	38,662 (8.1%)	13,905 (6.8%)	3.061 (9.5%)
Toronto/York	18,316 (43.1%)	105,789 (40.6%)	94,907 (38.3%)	22,163 (25.4%)	142,953 (30.0%)	53,043 (26.1%)	10,902 (33.9%)

**Figure 1.**
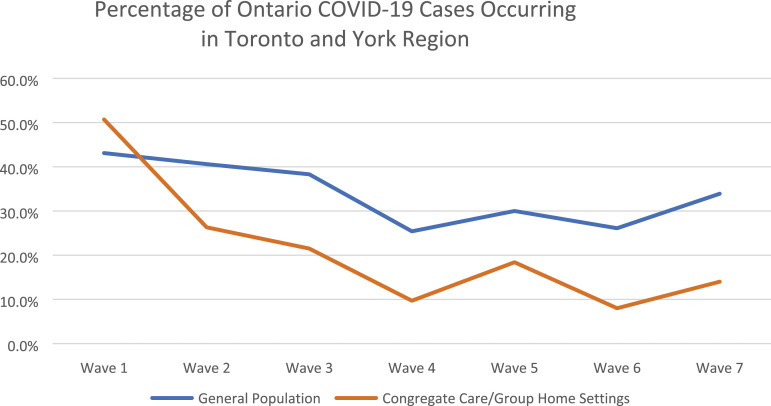
Percentage of Ontario COVID-19 cases occurring in Toronto and York regions.

This comparison is presented graphically in Figure 1. The contrasting patterns reflect the experience of IPAC program participants: very high infection rates in congregate care settings in Wave 1, followed by a rapid decline as IPAC measures were implemented, to comparatively low levels as measures were sustained. The data also illustrate the high percentage of Ontario cases in the early pandemic waves in Toronto and York Region, which include densely populated urban settings where infection control can be particularly difficult.

**Figure 2.**
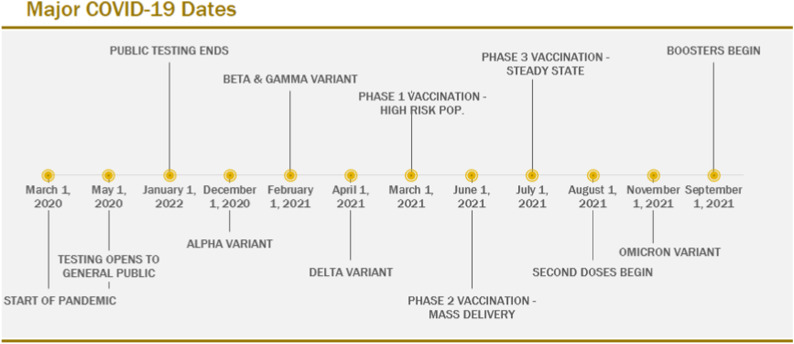
COVID-19 major timelines.

Data interpretation must consider multiple factors impacting on confirmed COVID-19 cases, including the arrival of new variants, the introduction of vaccines and changes to testing practices. Key dates in the evolution of the pandemic in Ontario are shown in Figure 2.

Although COVID-19 cases have subsided in congregate care settings, as in the broader population, many vulnerable clients remain at high risk for infection. Many IPAC practices introduced through the program remain in place. Education services and collaborative sessions continue at a reduced frequency. Sharing of IPAC information and expertise across organizations and sectors is ongoing.

Safehaven was nominated by Reena and received the 2021 OASIS Leadership Annual Award. Safehaven also received the 2021 National Children’s Healthcare Leaders Award for Excellence. The Healthcare Insurance Reciprocal of Canada (HIROC) recognized Safehaven’s efforts with the 2022 National Celebrating the Human Spirit Award. Safehaven received Accreditation with Exemplary Standing from Accreditation Canada in 2022, with staff reporting above-average workplace experiences.

## Conclusion

The response to the COVID-19 pandemic addressed major gaps in IPAC information, expertise, and resources available to congregate care settings. Subsequent IPAC program development has established a comprehensive system of IPAC services that are adapted to the needs of vulnerable populations, and consistent with best-practice clinical and public health standards.

The experience highlights the importance of recognizing diverse vulnerable populations as a priority in all pandemic prevention and response planning. IPAC strategies must identify and adapt to the needs of each vulnerable group, and to regional variations in access to healthcare resources and broader socio-economic factors. Ongoing funding to support IPAC programming for vulnerable individuals will be required.

The program initiated by Safehaven as an emergency response to the COVID-19 pandemic continues to evolve. In the early phases of the pandemic, participating organizations focused exclusively on responding to an unprecedented crisis. More recently, these organizations have been able to focus on issues such as cost reduction, technology optimization, and the efficient use of resources to ensure ongoing program viability. The program is now serving as a sustainable, cross-sector foundation to support continuous improvement in IPAC knowledge and practices in congregate care settings and prepare for future outbreaks.

Leadership has played a critically important role throughout this process. Leaders at all levels, from federal and provincial agencies, to the systems and organizations that provide care, to the teams that deliver essential services, demonstrated exceptional capacities to innovate, adapt to rapid change, support colleagues, and advocate for those in need. Specific principles and practices that have been shown to be most effective are highlighted below.

These efforts were initiated and continue within the context of the global response to the COVID-19 pandemic, including most importantly the focused mobilization of healthcare resources, the rapid development and deployment of COVID-19 vaccines, and the ongoing evolution of COVID-19 policies and protocols. These efforts will be essential in ensuring the health and safety of individuals in congregate care, as in the general population.

## Recommendations

The following recommendations have been developed to assist other organizations, sectors, and communities in preparing for and responding to future outbreaks of infectious disease.

• **Adopt a highly collaborative response, building partnerships within and across sectors, and working closely with experts and the appropriate authorities.** At the onset of the pandemic, Safehaven reached out immediately to DS sector agencies to share information, to area hospitals to access IPAC expertise, and to public health agencies and the Ontario Ministry of Health to advocate for support and ensure consistency with available best-practice standards. These collaborative processes continue today, and have played a key role in securing essential resources and identifying congregate care settings as a public health priority.
• **Ensure that all education and communication programs and materials are adapted to meet the specific needs of target audiences.** Much of the work required in the early phases of the emergency response involved translation of available IPAC information into practical terms and formats suitable for non-clinical DS sector staff. IPAC collaborative sessions focused specifically on the challenges confronted by DS sector leaders. Careful customization of all education and communication efforts is essential for efficient learning and rapid implementation of emergency response measures.
• **Respond to the emergency while also anticipating future needs.** Emergency management requires the best possible responses to real-time events, while simultaneously anticipating and preparing for upcoming needs. The capacity of leadership teams to be forward thinking and to adopt a systems perspective supports collaboration and a rapid response.
• **Apply flexibility and creativity to adapt to an evolving crisis.** In the early waves of the COVID-19 pandemic, vaccines were not yet available and public health standards were evolving. In this context, program leaders focused initially on well-established infection control basics such as hand hygiene. Increasingly effective and targeted solutions were incorporated as they became available, always in consultation with clinical experts and approval authorities. As in many crisis situations, the response required numerous innovations. The mobile unit referenced above, for example, was created because there was no other way to quickly place IPAC expertise in congregate care settings.
• **Provide essential staff supports.** Safehaven employs and encourages practices such as regular rounding and daily huddles to keep all staff connected and to enable leaders to identify early signs of fatigue and provide breaks and supports as needed. A leadership approach grounded in compassion and focused on the unique needs and capacities of each staff member is essential for sustainable progress, during and after the initial emergency.
• **Adapt emergency measures as the crisis subsides, and identify opportunities for sustainable change.** As the numbers of COVID-19 cases, severe illnesses, and deaths have subsided, practices implemented during the emergency have been adjusted. Some infection prevention and control measures have been relaxed, while others, such as masking for vulnerable clients, remain in place. DS sector leaders are using the experience and knowledge gained to improve prevention of all infectious diseases, and readiness for future pandemics. While the COVID-19 pandemic is no longer considered an emergency, sustainable IPAC practices to protect vulnerable clients must continue. The sector recognizes the importance of embracing practices that keep clients safe and healthy.
• **Advocate on behalf of vulnerable and/or underserved groups and individuals.** Safehaven and Reena have been relentless advocates for those with developmental disabilities and complex care needs throughout the COVID-19 pandemic. Advocacy has played an essential role in securing resources and changing public health policies and programs to ensure that the DS sector and other vulnerable groups are included in pandemic prevention and response strategies, now and in the future.

## Notes

1. Safehaven. We Belong. Accessed 06/07/2023. www.safehaven.to

2. Lunsky Y, Durbin A, Balogh R, et al. COVID-19 positivity rates, hospitalizations, and mortality of adults with and without intellectual and developmental disabilities in Ontario, Canada. *Disability and Health Journal*. 2021. https://doi.org/10.1016/j.dhjo.2021.101174

3. Gilbert J, Croson W, Vitkin, N. Safehaven IPAC Program Evaluation*.* Julie Gilbert Consulting; June 15, 2022

4. Public Health Ontario. Response to scientific/technical request: cases, outbreaks, and outbreak-associated cases in Safehaven specified settings from March 1, 2020 to July 16, 2022. Requestor: Safehaven. Request date: 02/24/2022. Response date: 07/27/2022. Data limitations identified by Public Health Ontario include:

- A rapid increase in cases starting in December 2021 led to changes in testing policies, issues with testing availability, and delays in data entry, most likely resulting in underestimated cases.

- Mandatory mass testing in Ontario ending in early 2022.

- Some data limitations contributed specifically to underrepresentation of cases in congregate care/group home settings. These include reporting delays and variations in data entry processes across public health units. Identification of cases relied largely on self-reporting of residence or employment in these settings as a risk factor, contributing to underreporting.

